# Comparison of clean catch and bag urine using LC–MS/MS proteomics in infants

**DOI:** 10.1007/s00467-023-06098-3

**Published:** 2023-07-31

**Authors:** Richard Klaus, Teresa K. Barth, Axel Imhof, Franziska Thalmeier, Bärbel Lange-Sperandio

**Affiliations:** 1 Division of Pediatric Nephrology, Department of Pediatrics, Dr. V. Hauner Children’s Hospital, Ludwig-Maximilians University, Lindwurmstraße 4, 80337 Munich, Germany; 2 Faculty of Medicine, Biomedical Center, Protein Analysis Unit, Ludwig-Maximilians University, Planegg-Martinsried, Munich, Germany

**Keywords:** Urinary proteomics, Urine collection, Liquid chromatography-mass spectrometry, Chronic kidney disease, Infants

## Abstract

**Background:**

Urinary proteomics identifies the totality of urinary proteins and can therefore help in getting an early and precise diagnosis of various pathological processes in the kidneys. In infants, non-invasive urine collection is most commonly accomplished with a urine bag or clean catch. The influence of those two collection methods on urinary proteomics was assessed in this study.

**Methods:**

Thirty-two urine samples were collected in infants using urine bag and clean catch within 24 h. Nine boys and seven girls with a mean age of 4.3 ± 2.9 months were included (5 × post-pyelonephritis, 10 × non-kidney disease, 1 × chronic kidney disease (CKD)). Liquid chromatography-mass spectrometry (LC–MS/MS) was performed in data-independent acquisition (DIA) mode. Protein identification and quantification were achieved using Spectronaut.

**Results:**

A total of 1454 urinary proteins were detected. Albumin and α-1-microglobulin were detected the most. The 18 top-abundant proteins accounted for 50% of total abundance. The number of proteins was slightly, but insignificantly higher in clean catch (957 ± 245) than in bag urine (876 ± 255). The median intensity was 1.2 × higher in the clean catch. Overall, differential detection of proteins was 29% between the collection methods; however, it diminished to 3% in the 96 top-abundant proteins. Pearson’s correlation coefficient was 0.81 ± 0.11, demonstrating a high intraindividual correlation. A principal component analysis and a heat map showed clustering according to diagnoses and patients rather than to the collection method.

**Conclusion:**

Urinary proteomics shows a high correlation with minor variation in low-abundant proteins between the two urine collection methods. The biological characteristics overrule this variation.

**Graphical abstract:**
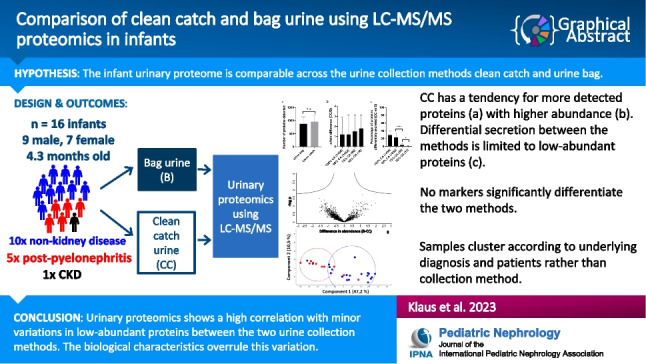
A higher resolution version of the Graphical abstract is available as Supplementary information.

**Supplementary Information:**

The online version contains supplementary material available at 10.1007/s00467-023-06098-3.

## Background

Urinary proteomics detects the totality of urinary proteins and quantifies their abundance [1–4]. In urinary proteomics, the proteins are identified from precursor peptides detected with liquid chromatography-mass spectrometry (LC–MS/MS). Those precursor peptides are artificially generated by trypsin digestion. Around 1500 proteins can be identified from those precursors within a single urine sample using software-based in silico-generated databases [5]. This fundamentally differentiates proteomics from peptidomics, where endogenous peptides are analyzed. This analysis is most often currently performed by coupling of capillary electrophoresis to mass spectrometry (CE-MS). The majority of the urinary proteins originate from the kidneys and urinary tract [1] and therefore characterize processes within the kidneys and urinary tract. Hence, urinary proteomics has the potential to revolutionize non-invasive diagnostics. Possible applications are diagnosis of a kidney disease, stratification, and therapy guidance in a known kidney disease [1, 6].

Infants with congenital anomalies of the kidneys and urinary tract (CAKUT) [7, 8], preterm, and small for gestational age neonates [9] are high-risk groups for CKD and could greatly benefit from earlier identification of relevant CKD risk. This risk identification could result in a better-timed follow-up and earlier, more specific therapy. Non-invasive diagnostics such as urine analysis is especially interesting for infants, because even minimally invasive diagnostics such as blood draws or IV line insertions for application of contrast agents are often a threat to the child and would be desirable to avoid.

However, urine collection is challenging in non-toilet trained infants and the collection methods are heterogeneous. Urine in infants can be collected with either non-invasive (bag urine, clean catch, or rarely cotton balls placed in diapers in neonates), or invasive methods (catheterization or suprapubic puncture) [10]. In routine settings, clean catch urine and bag urine are the most commonly used methods in infants. For urinary biomarker search, it would be more practical to use bag urines. However, even though there is an abundance of knowledge on how bag urine and clean catch affect diagnostics of urinary tract infections and routine parameters [11, 12], no comparative analysis on their effect on urinary proteomics exists.

We hypothesize that the urinary proteome is comparable across urine collection methods for the individual infant, but that modification of the proteome due to proteolysis might occur in the urine bag samples. This is the first study to assess the influence of the two collection methods on the urinary proteome in infants. We aim to bring the powerful tool of urinary proteomics one step closer to infants with kidney disease.

## Materials and methods

### Patient characteristics

Urine samples were collected from inpatient infants at a single tertiary pediatric hospital after informed written consent from parents. Thirty-two urine samples from 16 patients were collected, nine from boys and seven from girls, and the mean age was 4.3 ± 2.9 months. Five patients had a recent, successfully treated pyelonephritis (post-pyelonephritis group). All of these 5 patients had at least 5 days of intravenous antibiotic treatment, absence of fever > 48 h, and a negative dipstick for leukocyturia. Nine infants had non-kidney disease (3 × RSV bronchiolitis, 1 × cystic fibrosis, 1 × gastroenteritis, 2 × feeding problems, 1 × upper GI bleeding, and 1 × inborn error of metabolism without acute crisis). One patient with proteinuric CKD due to perinatal bilateral renal vein thrombosis was included. None of the patients had clinical signs of an active bacterial infection. The local ethics committee gave a positive vote on the study protocol (22–0538).

### Urine sample collection and preparation

All urine samples were collected in our hospital from inpatient infants. Genitalia were cleaned with isotonic saline prior to urine collection for both methods and medical staff were wearing gloves. Braun Urinocol (article number 227550A for boys, article number 227560A for girls) was used for urine bag collection and clean catch urine was performed with a 70-ml sterile urine beaker (Sarstedt, article number 75.9922.745). Medical staff at our hospital are trained in obtaining clean catch urine samples, since it is the most commonly used method to exclude urinary tract infection in infants in our hospital. If the parents collected the sample, they were instructed by medical staff on how to perform a clean catch. The clean catch urine sample was immediately transferred from the urine beaker to a 10-ml urine monovette (Sarstedt, article number 10.252) and immediately frozen to − 20 °C. Time from voiding to freezing for the clean catch samples was therefore less than 5 min.

For urine bag collection, medical staff placed the urine bag and parents were instructed to check for urine at least every 45 min. If the bag contained urine, staff were notified by the parents and the sample was immediately transferred to a 10-ml urine monovette (Sarstedt, article number 10.252) and frozen to − 20 °C. Therefore, the maximum time between voiding and freezing for the bag urine was 50 min, but unknown for each individual sample. The maximum time period between the collections of the two urine samples was 24 h. The maximum storage time at − 20 °C was 3 months. If exceeded, urine was transferred to − 80 °C. All samples were thawed, processed, and analyzed at the same time as described below.

After thawing on ice, urine samples were centrifuged at 12,000 g at 4 °C for 5 min and supernatants transferred to a new plate. No protease inhibitors were used. A BCA assay (kit 23,225 from Thermo Scientific, Pierce) was performed, and 50 µg protein of each sample was prepared for analysis using the iST kit with SP3 add-on (PreOmics GmbH, Martinsried, Germany). Briefly, proteins were bound to magnetic beads, washed, and resuspended in lysis buffer. Proteins were digested with trypsin and Lys-C for 3 h and peptides subsequently cleaned up (including WASH0 specific for urine samples), eluted, dried, and resuspended in 50-µl LC-LOAD buffer.

### Liquid chromatography-mass spectrometry proteomics

For LC–MS/MS analysis, trypsin-digested peptides were loaded on Evotip Pure tips (Evosep, Odense, Denmark) and analyzed on the Evosep One system using EV-1137 column (15 cm × 150 µm, 1.5-µm beads) and EV-1086 emitter (stainless steel, 30 µm ID) running the 30 samples per day method. The column was heated to 40 °C using a column oven (PRSO-V2-PS, Sonation GmbH, Biberach, Germany). Coupling to the Exploris 480 mass spectrometer was done with the Nanospray Flex Ion source (Thermo Fisher Scientific) with 2-kV spray voltage and 275 °C ion transfer tube temperature. The MS was operated in data-independent acquisition (DIA) mode with the following settings: full scan resolution 120,000, scan range 380–980 m/z, normalized AGC target 300% with maximum injection time of 100 ms. DIA scans were obtained from a precursor mass range of 380–980 m/z at an isolation width of 20 m/z per window with 1 m/z window overlap, resulting in 30 windows. Collision energy was 30%, normalized AGC target 3000% with maximum injection time mode set to auto. Orbitrap resolution was 30,000 and data acquired in centroid mode. Samples were analyzed in a randomized sequence.

### Protein identification

Data analysis was carried out using Spectronaut software (Biognosys) version 15.6.211220.50606. Data filtering was *q*-value sparse, and no normalization or imputation was chosen. Protein identification was based on 1 or 2 tryptic peptides in 37–52% of proteins in the individual samples. Protein identification was done with an in silico-generated database with trypsin as the default enzyme. The Sprot human database was downloaded within Spectronaut (from April 2021, 20,394 protein entries). The exported data file was opened and analyzed in Perseus version 2.0.9.0.

## Results

Thirty-two urine samples were collected in infants using urine bag and clean catch within 24 h. Nine boys and seven girls with a mean age of 4.3 ± 2.9 months were included (5 × post-pyelonephritis, 10 × non-kidney disease, 1 × CKD). All patients were Caucasian and European.

### Few proteins make up the majority of the total urinary protein abundance and about half of the most abundant proteins are shared with the healthy adult urinary proteome

**Fig. 1:**
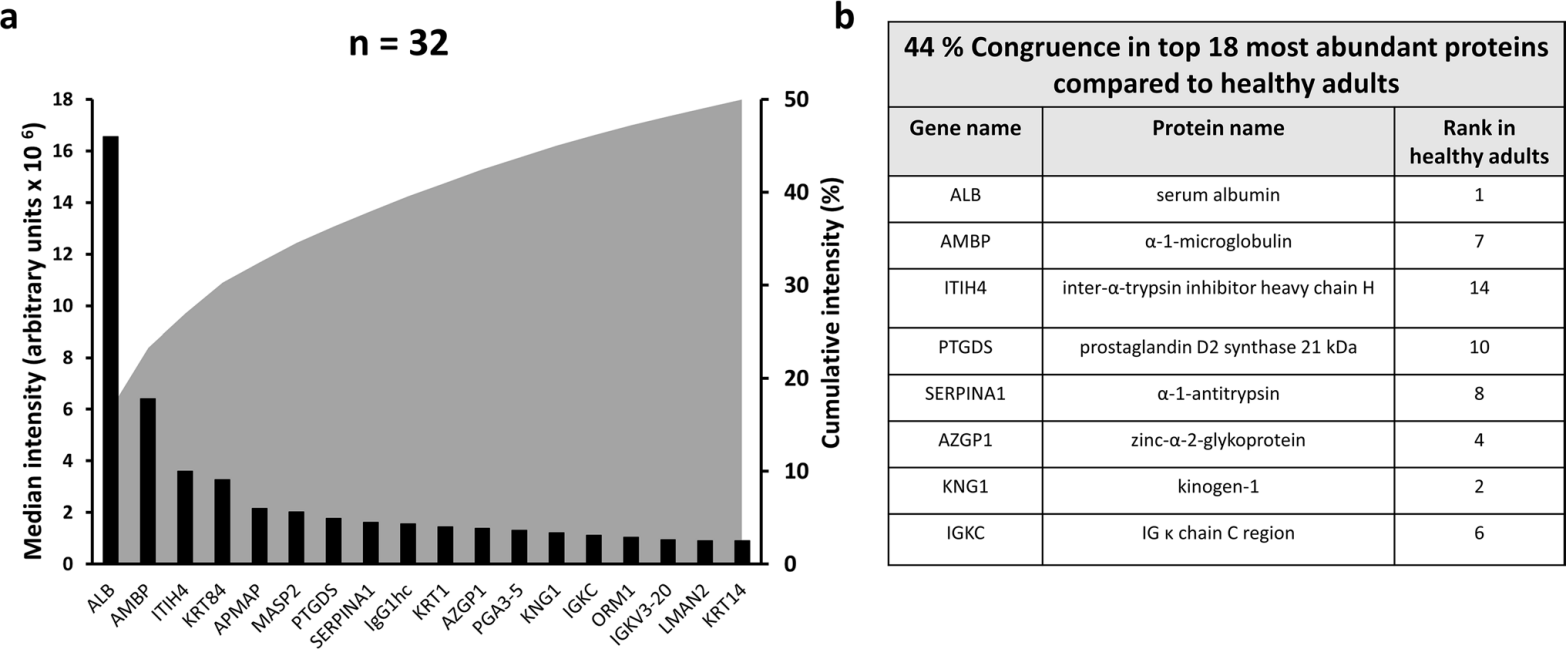
The 18 most abundant proteins make up 50% of cumulative abundance and 44% of them are shared with an adult reference proteome (*n* = 32 urine samples from 16 patients). **a** The black bars show the median abundance of the 18 most abundant proteins (median with interquartile range). The gray area represents the cumulative abundance of the proteins. **b** List of congruent proteins when compared with the top most abundant proteins from an adult standard cohort [13]

A total of 1454 urinary proteins were identified in 32 samples. The total number of proteins detected in a single patient in both urine bag and clean catch urine together was 1068 ± 195 (Table 1). A cumulative abundance of 50% was reached with the first 18 most abundant proteins (sorted according to median abundance) (Fig. 1a). The most abundant protein was albumin, with a 2.6-fold higher abundance than the second most abundant protein α-1-microglobulin/bikunin precursor (AMBP), which had a 1.8-fold higher abundance than the next protein. Eight of the top 18 proteins (44%) were also listed in the top 18 proteins in a standard urinary proteome in adults [13]. A search for bacterial proteins revealed no relevant difference in the samples.

**Table 1:** Number of identified proteins per patient and intraindividual variability

Patient	Proteins in B	Proteins in CC	Total proteins	Only detectable in either B or CC	x-fold intensity if detected in both (CC/B)	Percentage differentially secreted between B and CC
1	1095	1134	1181	133	1.3	11
2	1002	780	1030	278	0.9	27
3	521	530	637	223	0.8	35
4	954	800	993	232	0.5	23
5	474	993	1004	541	5.2	54
6	668	1208	1279	682	2.8	53
7	1070	1081	1146	141	1.1	12
8	854	487	859	377	0.5	44
9	682	577	746	233	0.9	31
10	761	1211	1221	470	4.9	38
11	1244	1125	1280	191	1.1	15
12	746	1187	1196	459	7.4	38
13	1086	1122	1187	166	1.4	14
14	479	843	860	398	3.4	46
15	1246	1194	1287	134	0.8	10
16	1126	1041	1177	187	1.4	16
**Mean ± SD**	**876 ± 255**	**957 ± 245**	**1068 ± 195**	**303 ± 161**		
**Median with interquartile range**					**1.2 (0.9–2.9)**	**29 (15–40)**

*SD*, standard deviation; *CC*, clean catch; *B*, bag urine

### The number of proteins and their abundance is higher in clean catch urine and low-abundant proteins are differentially secreted between the methods

**Fig. 2:**
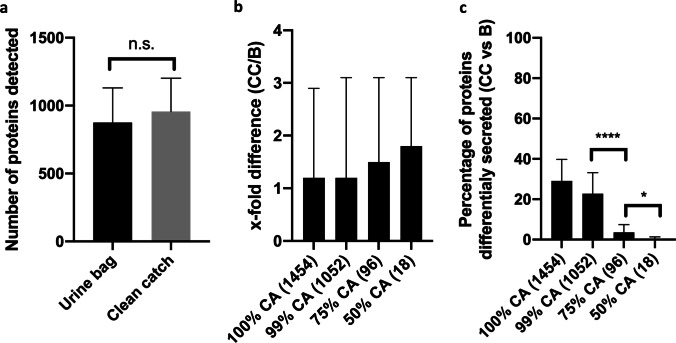
**a**, **b** Clean catch urine has a tendency toward more detected proteins with higher abundance; **c** low-abundant proteins have an intraindividual differential secretion between the two collection methods. **a** The bars show the number of detected proteins in bag collection (black) and clean catch collection (gray) (mean ± SD). **b** The x-fold intensity (clean catch/bag urine) between the intraindividual bag urine and clean catch samples (both median with interquartile range) in 100% of detected proteins and top most abundant proteins, which make up 99%, 75%, and 50% of cumulative abundance (CA), respectively, is shown. The number of proteins in the respective cumulative groups is listed in brackets. **c** The percentage of differentially secreted proteins between the intraindividual clean catch urine (CC) and bag urine (B) samples (both median with interquartile range) in 100% of detected proteins and top most abundant proteins, which make up 99%, 75%, and 50% of cumulative abundance (CA), respectively, is shown. Statistical significance was tested with unpaired *t*-test (****p < 0.001, *p < 0.05)

The mean number of detected proteins in urine bag collection was 876 ± 255 (range: 479–246) and therefore slightly lower than in collection with clean catch (957 ± 245, range: 487–1211) (Table 1). However, no significant difference in protein number between the collection methods was found (Fig. 2a).

Not only the number of proteins but also their abundance was higher in the clean catch samples. The x-fold difference in abundance in proteins detected in both samples favored the clean catch urine among all groups of cumulative abundance with 1.2 × in all proteins and 1.2 × , 1.5 × , and 1.8 × in the groups with 99%, 75%, and 50% cumulative abundance, respectively (Fig. 2b).

Taking 100% of proteins in a single patient into account, 29% of proteins were found only in one of the urine samples (either urine bag or clean catch of the individual patient) (Table 1 and graphically displayed in Fig. 2c). However, this relatively high number significantly diminished when looking into the subgroup proteins stratified by cumulative abundance (Fig. 2c). In the proteins accounting for 100% (1454 proteins) and 99% (1052 proteins) of cumulative abundance, the percentage of differentially secreted proteins between clean catch urine and urine bag was 29% and 23%, respectively. However, in the 96 most abundant proteins accounting for 75% of cumulative abundance, only 3% (*p* < 0.001) were differentially secreted between the samples. Differential secretion dropped to 0% for the top 18 most abundant proteins accounting for 50% of cumulative abundance (*p* = 0.0225). Therefore, differential secretion was a phenomenon limited to low-abundant proteins (Fig. 2c).

### There is a high intraindividual correlation between urine bag and clean catch urine, with no markers significantly differentiating the two methods

**Fig. 3:**
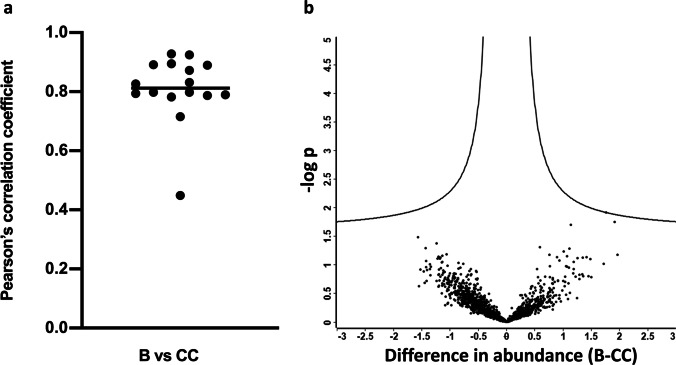
There is a high intraindividual correlation between urine bag and clean catch, with no significant markers differentiating the two methods. **a** Each dot represents a single patient. Pearson correlation coefficient between urine bag (B) and clean catch (CC) collection was calculated for all proteins within the samples of the patient with Perseus. Mean correlation coefficient was 0.81 ± 0.11 (mean ± SD). A perfect correlation would have a value of 1. **b** A volcano plot to identify proteins, which are different between bag urine (B) and clean catch urine (CC). Each dot represents a single protein. On the x axis, the difference between the two groups is plotted (B-CC). The y axis plots the statistical significance as − log p. Points above the curved lines would represent markers significantly different between the two collection methods

To determine if intraindividual samples are alike between the collection methods, Pearson’s correlation coefficient was calculated (Fig. 3a). Mean intraindividual Pearson’s correlation coefficient between urine bag and clean catch was 0.81 ± 0.11 (mean ± SD) (Fig. 3a), demonstrating a high intraindividual correlation. There were no markers differentiating the urine collection methods. In a volcano plot, no proteins were identified to significantly discriminate between urine bag collection and clean catch collection (Fig. 3b). The identified proteins of both groups (urine bag and clean catch) scattered below statistical significance.

### Samples cluster according to patients and diagnosis rather than to collection method

**Fig. 4:**
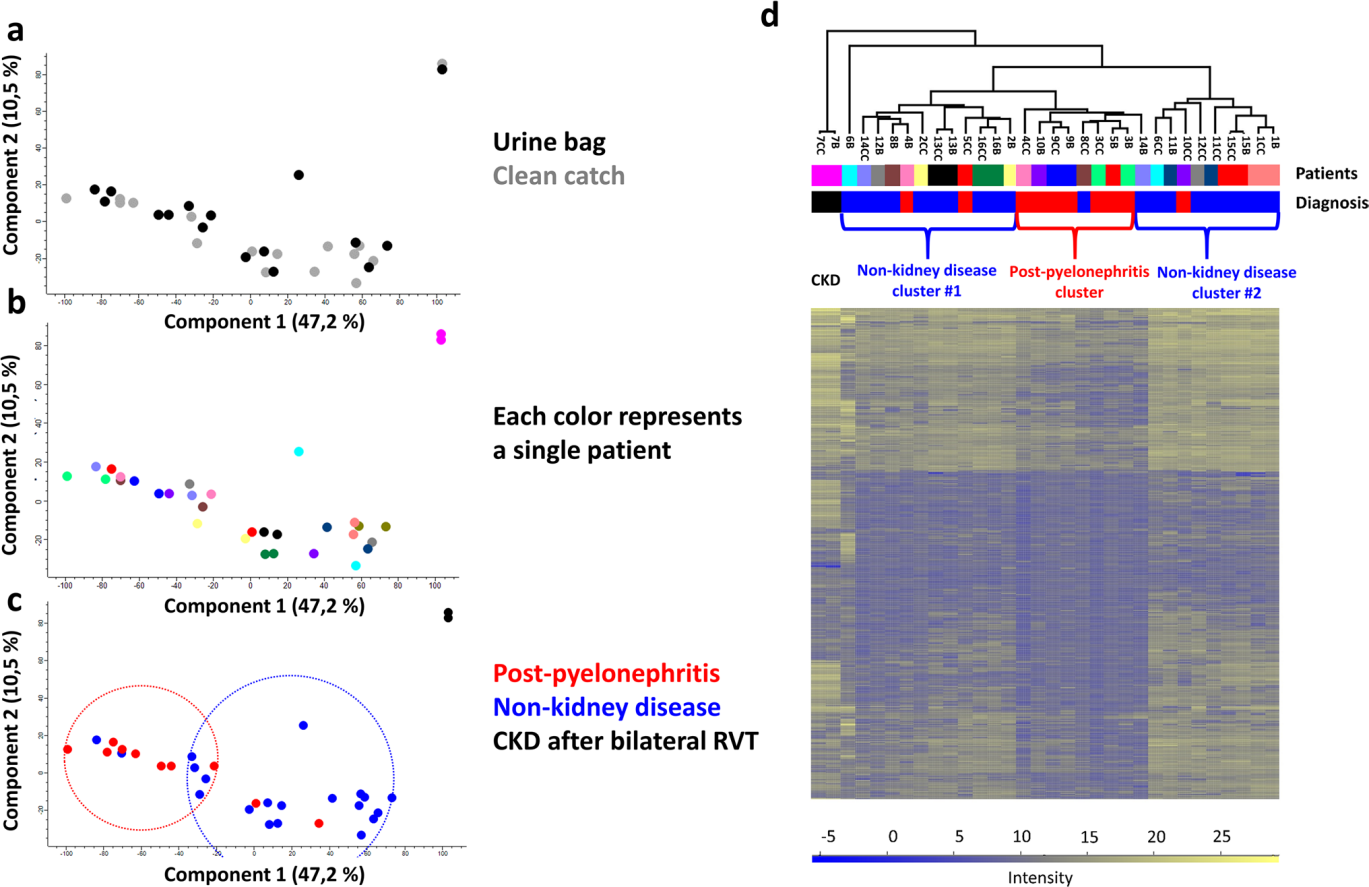
Samples cluster according to diagnosis and patient rather than according to the collection method. **a**–**c** Principal component analysis (PCA) of the samples. Each dot represents a different sample. The closer the two samples lie together on this 2-D-map of the principal components, the more they are alike. Principal component 1 (x axis) is accountable for 47.2% of the variability of the samples and principal component 2 is accountable for 10.5% of the variability of the samples (y axis). **a** Color coding is according to collection method, black is bag urine and gray is clean catch urine; **b** patients; **c** every single color represents a patient and diagnosis, red is the post-pyelonephritis group, blue is the non-kidney disease group, and black is the patient with CKD. **d** Two-dimensional heat map with unsupervised hierarchical clustering. Patients are numbered from 1 to 16, B stands for bag urine and CC for clean catch urine. Samples are clustered due to similarity. Patients and diagnosis are color coded like in **b **and **c**

To analyze if the different urine samples cluster according to the collection method (Fig. 4a), to patients (Fig. 4b), or underlying diagnosis (Fig. 4c), a principal component analysis was performed. For the underlying diagnosis, patients were grouped into non-kidney disease and post-pyelonephritis groups. One single patient with CKD due to bilateral renal vein thrombosis was included to determine if a clinical outlier with relevant kidney disease would also appear as a proteomic outlier regardless of the collection method. Component 1 is accountable for 47.2% of the variability and component 2 is accountable for 10.5% of the variability of the samples. Patients do not cluster visually according to the urine collection method (Fig. 4a), but rather according to the patient (Fig. 4b) and best according to the underlying diagnosis (Fig. 4c). Unlike in other studies, no clustering according to the patients’ sex was observed in our samples (data not shown).

For a different representation of clustering, data was computed according to unsupervised two-dimensional hierarchical clustering using a 2D heat map (Fig. 4d). In the first level of clustering, the patient with CKD (patient 7) separated from all other patients with both his clean catch and his urine bag sample. The bag urine of patient 6 was also a complete outlier on cluster hierarchy level 2. On the fourth hierarchical level, 3 major clusters formed (2 for non-kidney disease (blue) and 1 for post-pyelonephritis group (red)). A total of 83% (30 of 36) clustered with patients from the same diagnosis group. On the last level of hierarchy, 33% of samples were directly neighboring the other sample from the same patient with the different urine collection method, demonstrating that these samples are most similar to each other.

## Discussion

Urinary proteomics is a promising tool to detect kidney processes earlier than conventional diagnostics such as microalbuminuria, serum-creatinine, and imaging studies. Infants at high risk for CKD might greatly benefit from a faster diagnosis and potentially more targeted therapy. In recent years, several approaches have been used to identify biomarkers with the omics-approaches including urinary proteomics, urinary peptidomics, and urinary metabolomics. Urinary proteomics was able to detect disease markers in IgA-nephropathy and IgA-vasculitis-nephritis [14], and even non-kidney diseases like respiratory diseases can be predicted in preterm neonates using urinary proteomics with LC–MS/MS [15, 16]. CE-MS peptidomics predicted the need for surgery in a small cohort in ureteropelvic junction obstruction (UPJO) [17–19], and long-term data from conservatively treated children with UPJO—although clinically healthy—showed a differential pathologic urinary peptidome indicating kidney remodeling [20]. In posterior urethral valves, peptidomics can predict the risk for CKD [21–23]. Urinary metabolomics was able to predict the need for surgery in 50 neonates with UPJO [24]. Diagnostic and predictive urine metabolomics fingerprints were identified in children with non-kidney disease such as spinal muscular atrophy [25]. All these findings—although to date not yet validated in larger multicenter studies—show the potential to identify biomarkers with omics-based diagnostics for kidney and non-kidney disease even in clinically asymptomatic children. Although some of the results are more than 10 years old, to date, no multicenter studies with bigger cohorts were able to validate those promising findings. This raises the question whether pre-analytical challenges such as contamination and unclear time-to-freeze in bag urine hinder the omics-based search for biomarkers. Consequently, this is the first study to assess the influence of the two most commonly used routine urine collection methods (bag urine and clean catch) on urinary proteomics in infants.

We identified 1454 proteins in 32 urine samples using both collection methods. Compared to our study, the few previous urinary proteomics studies in infants and newborns have identified a similar number of proteins or less (242–1498 per sample) with either catheterization, bag urine, or an unspecified collection method [15, 16, 26]. We identified albumin and α-1-microglobulin/bikunin precursor (AMBP) to be the most abundant proteins in infant urine. Albumin is the most abundant serum protein and does not pass the glomerular filtration barrier in a healthy kidney in great amounts. α-1-microglobulin is freely filtered and almost completely reabsorbed; increased secretion indicates tubular damage. The high abundance of albumin and α-1-microglobulin in our proteome analysis is congruent with the findings in healthy adults [13]. However, there is only a 44% congruence in most abundant proteins of our infant urinary proteome in comparison to healthy adults. This is similar to the only other study comparing the urinary proteome of infants and adults where 45% of 1584 proteins were commonly detected between 6 healthy infants and 6 healthy adults [26]. Froehlich et al. furthermore found that infant-specific proteins are involved in translation and transcription, cellular growth, and metabolic processes, while the adult-specific proteins are involved in immune responses and cell adhesion [26]. This change over age seems to be a common feature in urinary omics-diagnostics. A change over age has also been demonstrated in urinary peptidomics [19] and urinary metabolomics [27]. In addition to age, Shao et al. were able to show a clear clustering according to gender [28], which however was not seen in our study. Overall, it becomes apparent that the standard urinary proteome may even change within the pediatric age groups (e.g., preterm, newborn, infants, toddlers, and adolescents) and the sexes. Reference cohorts need to be characterized more precisely, in order to use the powerful tool of proteomics for the identification of biomarkers.

In comparison of the two analyzed collection methods, an interesting phenomenon observed was the tendency for a lower number of detected proteins and a lower intensity of proteins in the urine bag samples. Contamination of bag urine with bacteria or leukocytes is a well-known phenomenon in the analytics of infant urine for urinary tract infection [10, 29, 30]. A contamination would result in a higher number of detected proteins in case of cellular contamination (e.g., proteins specific for leukocytes or epithelial cells) and in the detection of bacterial proteins in case of bacterial contamination. In our study, we only found very few bacterial proteins with no difference between the collection methods and the overall number of detected proteins was lower in the bag urine. We hypothesize that the slightly lower number and abundance of detected proteins in the bag urine results from endogenic proteolysis occurring between voiding and detection of the filled urine bag [31]. In the infant, urine is exposed to endogenic proteases within the bladder for the duration of the voiding interval of 1 to 2 h [1, 32]. These proteases continue to minimally alter the urine in bag collection, because the urine is stored close to the infant’s body and stays approximately at body temperature. In our study, the maximum time the urine stayed in the bag was 45 min, resulting in an almost doubled time of endogenic protease exposure at body temperature in the worst case. Data from adults indicate that storage at room temperature for up to 4 h does not significantly change the number of proteins detected, but minimally decreases the number of detected proteins [33]. This is in line with our data showing a tendency for fewer proteins and lower abundancy in the urine bag sample. After cooling or freezing, the proteolytic activity of urinary protease activity can be neglected. Adult studies showed that the urinary proteome does not change significantly when urine was stored up to 3 days at 4 °C or up to 6 h at room temperature. Urine can be stored for several years at − 20 °C without significant alteration of its proteome [1, 34, 35]. We therefore chose to not use protease inhibitors, since proteolysis in the cooled or frozen urine seems negligible. Not using protease inhibitors for urinary proteomics has also been recommended by several authors [33, 36, 37].

Our study showed a minor variability between the two urine collection methods. One third of urinary proteins were differentially detected between the collection methods within the same individual within 24 h. This phenomenon was however limited to the multitude of low-abundant proteins. Highly abundant proteins showed no relevant differential secretion between the two collection methods. These findings might have relevant impact on the search for urinary biomarkers in infants. A urinary biomarker could either be completely absent in health and present only in disease or vice versa or be present in both health and disease, but in a different abundance (either increased or decreased). According to our results, mixing the urine collection methods in a potential study might result in a non-identification of a marker of the first type. If the intraindividual differential secretion of low-abundant proteins found in this study holds true, then the search for urinary biomarkers of the “absent-or-present” type would be limited to markers with a high abundance, where there is a high congruence between the methods. According to our findings, identification of a biomarker characterized by different abundance between health and disease could be difficult when collection methods are mixed, since the clean catch urine samples showed a higher abundance of the individual proteins. If the collection methods are mixed, the x-fold change due to biological processes would need to be high to outperform the variability due to the collection method. The use of a correction factor for abundance is theoretically possible but will introduce another variable to an already challenging search. It could therefore be expected that use of clean catch urine not only results in a lesser intraindividual variability due to lesser risk of contamination [10] but also in a better control of the time taken until the sample is frozen and therefore higher protein number and higher abundance of the individual proteins.

Data from adults show that our observed minor variability might not entirely result from the collection method but from intraindividual variation. This variation is likely caused by circadian rhythm, activity, diet, and metabolic or catabolic processes [1]. In midstream urine (which is comparable to clean catch) in adults, there was a coefficient of variation of 0.44 but intraindividual samples clustered perfectly according to the individual using LC–MS/MS [13]. Another study found coefficients of variation of 1.3–5.7% for the inter-day variability in CE-MS even in the top 4 most abundant peptides. Regardless of the minor differences between the urine collection methods, it is promising to see that our study is equivocal to other studies and shows that biological characteristics overrule the minor analytical and pre-analytical variations.

There are some limitations to our study. First, not all the possible methods of urine collection in infants were included in this study: The gold standard to obtain a sterile urine sample is invasive and would either be urinary catheterization or a suprapubic bladder puncture. Due to the invasive nature of those methods, they are limited to emergency situations to exclude urinary tract infections in infants and would be impractical for any proteomic biomarker study. The non-invasive clean catch sample may yield the same quality for urinary proteomics, since it was shown that it is non-inferior to catheterization in diagnostic use for urinary tract infection and routine clinical parameters [10, 38]. Secondly, urine collection by cotton balls was not included in our study, since it was shown to have negative bias on the routine parameters albumin and total protein amount in a recent study [12]. Thirdly, the inclusion of a CKD patient and the lack of healthy control infants may be another limitation to our study.

Our results clearly demonstrate that there are some issues that need to be addressed before urinary proteomics in infants can become a robust and clinically useful tool. First, age-specific and gender-specific reference proteomes need to be established. Second, superiority in intraindividual variation of clean catch should be demonstrated. Finally, further methods of urine collections and their impact on urinary proteomics should be analyzed.

## Conclusion

Urinary proteome analysis shows a high intraindividual correlation between urine bag and clean catch collection in infants. The biological characteristics overrule the minor variability observed in low-abundant proteins. Nevertheless, it would be ideal to use the same standardized collection method in the search for biomarkers. Clean catch urine may be preferred due to a better control of pre-analytical variability and higher protein yield. Further studies on urinary proteomics in infants are needed to draw final conclusions.

### Supplementary Information

Below is the link to the electronic supplementary material.

Graphical Abstract (PPTX 275 KB)

## Data Availability

All data can be made available upon reasonable request to the corresponding editor.

## Abbreviations

CAKUT: Congenital anomalies of the kidneys and urinary tract
CE-MS: Capillary electrophoresis-mass spectrometry
CKD: Chronic kidney disease
DIA: Data-independent acquisition
LC–MS/MS: Liquid chromatography-mass spectrometry
UPJO: Ureteropelvic junction obstruction
