# Supplementary material for: Comparison of clean catch and bag urine using LC–MS/MS proteomics in infants
Source: Pediatr Nephrol. 2023 Jul 31;39(1):203–12. doi: 10.1007/s00467-023-06098-3 (PMC10673958; doi:10.1007/s00467-023-06098-3)
Supplement: Supplementary file 1 — Graphical Abstract (PPTX 275 KB) [file 467_2023_6098_MOESM1_ESM.pptx]

## Slide 1
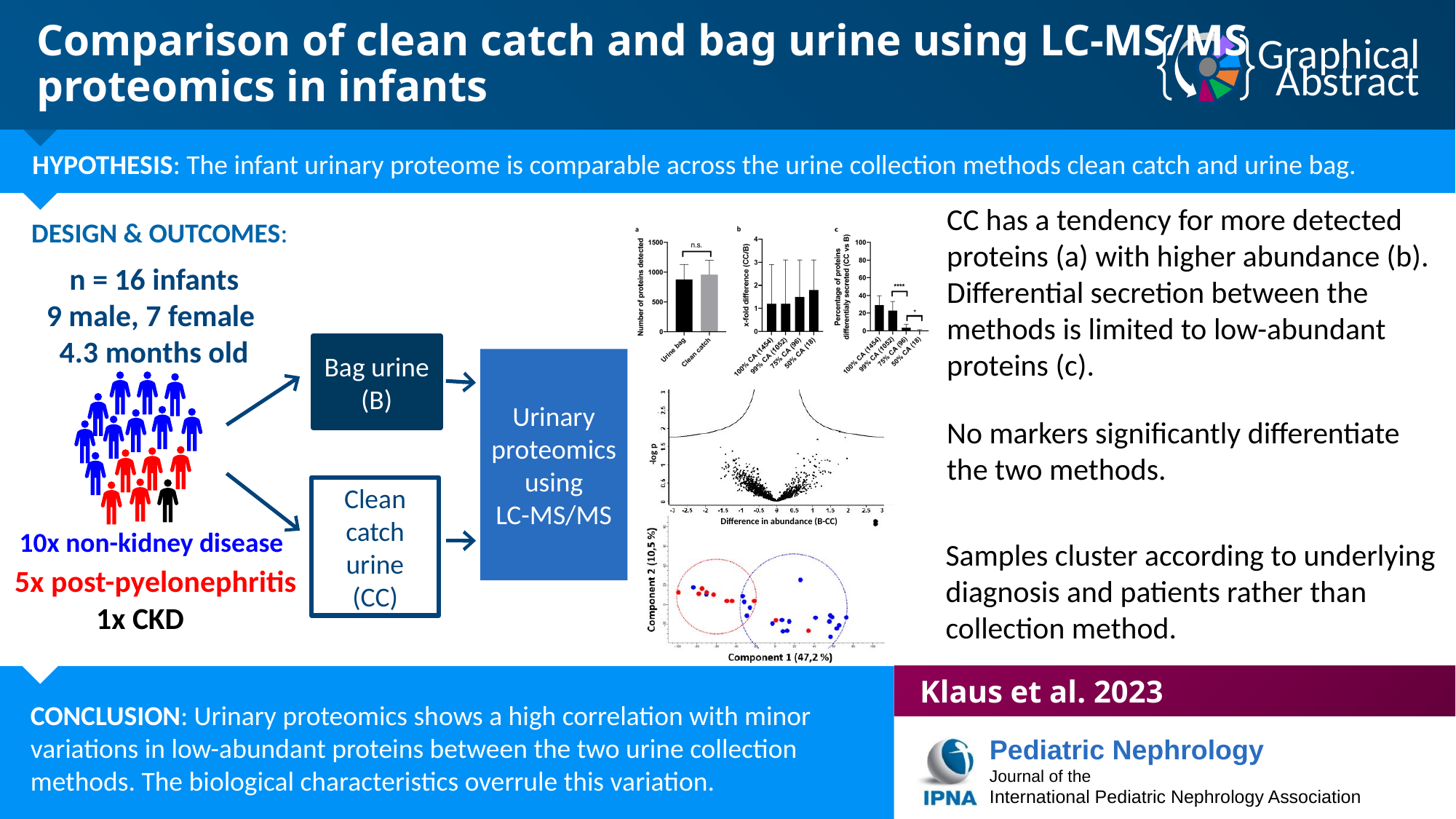

Comparison of clean catch and bag urine using LC-MS/MS
proteomics in infants
HYPOTHESIS: The infant urinary proteome is comparable across the urine collection methods clean catch and urine bag.
CC has a tendency for more detected proteins (a) with higher abundance (b).
Differential secretion between the methods is limited to low-abundant proteins (c).
DESIGN & OUTCOMES:
n = 16 infants
9 male, 7 female
4.3 months old
Bag urine
(B)
Urinary
proteomics
using
LC-MS/MS
No markers significantly differentiate the two methods.
-log p
Clean catch urine (CC)
Difference in abundance (B-CC)
10x non-kidney disease
Samples cluster according to underlying diagnosis and patients rather than collection method.
5x post-pyelonephritis
1x CKD
Klaus et al. 2023
CONCLUSION: Urinary proteomics shows a high correlation with minor variations in low-abundant proteins between the two urine collection methods. The biological characteristics overrule this variation.
